# Evaluate the effectiveness of cognitive stimulation and physiological dynamization on sleep inertia

**DOI:** 10.3389/fphys.2026.1855408

**Published:** 2026-08-24

**Authors:** Vincent Beauchamps, Fabien Sauvet, Stanislas Boyer, Federico Nemmi, Florence Buratto

**Affiliations:** 1 Université Paris Cité, VIFASOM, Paris, France; 2 Institut de recherche biomédicale des armées (IRBA), Brétigny sur Orge, France; 3 Human Design Group, Toulouse, France; 4 AIRBUS Commercial Aircraft, Flight Operations & Training Policies Dptmt, Blagnac, France

**Keywords:** cognitive performance, cognitive stimulation, dynamization, psychomotor vigilance task, sleep inertia, sleepiness

## Abstract

**Introduction:**

Sleep inertia, characterized by impaired cognitive and psychomotor performance post-waking, poses a significant safety concern in operational settings, particularly when exacerbated by prior sleep loss. Existing reactive countermeasures demonstrate limited efficacy within the critical initial minutes following awakening. This study aimed to evaluate the effectiveness of two reactive interventions—cognitive stimulation and physiological dynamization —in mitigating sleep inertia, examining their impact after both a 9-hour time in bed baseline night and a 3-hour recovery night following total sleep deprivation.

**Methods:**

Forty-two healthy participants were randomized into Control, Cognitive Stimulation, or Dynamization (respiratory and muscular exercises) groups, performing a 5-minute countermeasure immediately upon awakening. Primary outcomes included Psychomotor Vigilance Test reaction times and attentional lapses, Karolinska Sleepiness Scale, and the Operational Assessment of Sleep Inertia State, measured at predefined intervals of 5, 20, 35, 40, 55 minutes post awakening. In the absence of a pre-protocol, true rested baseline, the 55 minutes measure was taken as the within-session reference point.

**Resutls:**

We confirmed that sleep inertia was significantly more pronounced following the 3-hour recovery night. Crucially, physiological dynamization significantly reduced sleep inertia within the first 20 minutes post-awakening, evidenced by lower reaction times (-49 ms, Cohen’d=0.81, p=0.02) and lapses (-2.3 lapses, d=0.71, p=0.02) at the Psychomotor Vigilance Task, and decreased subjective sleep inertia scores compared to the control group. Cognitive stimulation also conferred some benefits, notably reducing lapses and subjective sleepiness inertia scores, though its effect was not as consistent as that of dynamization. Participants in the cognitive stimulation group showed higher subjective and objective measures of sleep inertia after waking up, while no such significant differences were observed for the dynamization group.

**Conclusion:**

These findings indicate that physiological dynamization, incorporating brief respiratory and muscular exercises, is a highly promising reactive countermeasure for rapidly dissipating sleep inertia. Implementing such interventions post-awakening holds substantial potential value for enhancing immediate performance, though field studies are required to validate operational implications in demanding professional environments.

## 1 Introduction

Sleep inertia is the paradoxical phenomenon consisting in a period of impaired cognitive performance and grogginess, experienced after waking, that dissipates as time awake increases (Hilditch et al., 2017; Hilditch and McHill, 2019; Trotti, 2017). This impairment can last from a few minutes to several hours (Achermann et al., 1995; Dawson et al., 2021; Ferrara and De Gennaro, 2000; Hilditch and McHill, 2019; Jewett et al., 1999).

Sleep inertia may affect different cognitive (e.g. processing speed, working memory, decision making) and psychomotor (e.g., orientation, postural stability, motor coordination) functions (Bruck and Pisani, 1999; Romyn et al., 2022; Santhi et al., 2013; Wertz et al., 2006). Its kinetic (i.e., dissipation rate) can thus be assessed by performance on cognitive or behavioral tasks, and via self-report measures of alertness, sleepiness or fatigue (Achermann et al., 1995; Dinges et al., 1985; Evangelista et al., 2022; Jewett et al., 1999; Trotti et al., 2022; Sauvet et al., 2024), or with electroencephalogram-recordings of brain activity upon awakening (Gorgoni et al., 2015; Marzano et al., 2010; Tassi et al., 2006a).

Despite its transient duration, sleep inertia is a significant source of cognitive and operational performance impairment (Tassi and Muzet, 2000; Trotti et al., 2022; Wertz et al., 2006), and has been identified as a contributing factor in critical real-world incidents. Empirical evidence has implicated sleep inertia in several major accidents and operational failures (Armentrout et al., 2006; Marine Accident Investigation Branch, 2015; National Transportation Safety Board, 2021; NSW, 2023, Sauvet et al., 2024). Within the aviation sector specifically, episodes of sleep inertia have resulted in operational incidents and accidents attributable to impaired decision-making by flight crew members emerging from controlled in-flight rest periods (Air India, 2016; Transportation Safety Board of Canada, 2011). Note that performance impairments observed during sleep inertia can be exacerbated by prior sleep loss and circadian disorders (such as jet lag) (Dinges et al., 1985; Hilditch, Centofanti, et al., 2016; Hilditch et al., 2017; Hilditch and Fischer, 2023), two conditions that are particularly common in aircraft pilots.

Strategies to mitigate the effects of sleep inertia are especially important in fields that demand high cognitive performance despite restricted sleep, such as medicine, the military, and aviation. Given the safety risk that sleep inertia presents, several studies have investigated potential countermeasures to minimize the duration and severity of sleep inertia. Proactive countermeasures (i.e., countermeasures performed prior to waking), such as coffee consumption prior to sleep (Centofanti et al., 2020), have been shown to successfully eliminate sleep inertia after 2-h naps (Van Dongen et al., 2001). Other studies have tried to modulate nap durations to reduce fatigue while minimizing SI, finding that a controlled 10 minutes nap could reduce fatigue effect while minimizing sleep inertia after waking, but 30 minutes nap could not (Hilditch et al., 2016b, 2017). However, such strategies may not be optimal, or even feasible, for on-call workers whose awakenings are unpredictable.

Reactive countermeasures to sleep inertia (i.e., countermeasures implemented upon waking) may thus be more suitable for on-call contexts. Several reactive countermeasures have been empirically tested, such as caffeine ingestion upon waking (Hayashi et al., 2003; Newman et al., 2013), light exposure (Hayashi et al., 2003; Santhi et al., 2013), sound manipulation (Hayashi et al., 2004; Tassi et al., 1992), body temperature manipulation (Krauchi et al., 2006), and face washing (Hayashi et al., 2003). To date, there is overall limited evidence that these countermeasures can successfully dissipate sleep inertia within 15 min post-waking (Hilditch et al., 2016a). Since some on-call roles require a response within minutes (e.g., medical emergency personnel, firefighters, pilots), countermeasures that do not meaningfully improve performance within a very short time (i.e., minutes) cannot be relied upon. As a result, further research into reactive countermeasures that may dissipate sleep inertia within the critical first minutes after waking is needed.

A previous study has suggested that cognitive stimulation may reduce the intensity of sleep inertia (Wörle et al., 2020). From a theoretical standpoint, cognitive stimulation engaging the brain in tasks that require cognitive effort, such as logical reasoning or memory tasks, may be particularly effective in this regard (Harrison and Horne, 2000; Killgore, 2010; Marzano et al., 2010). These cognitive activities may activate regions of the brain involved in alertness and executive functions, such as frontal regions, potentially accelerating the transition from a sleep state to wakefulness (Harrison and Horne, 2000). However, while cognitive stimulation has been widely studied in the context of healthy aging (Gómez-Soria et al., 2023) and dementia (Cao et al., 2023), empirical studies on its effectiveness against sleep inertia are lacking.

Beyond cognitive stimulation, interventions that involve respiratory and physical movement, may also be pertinent to decrease sleep inertia (Dawson et al., 2021; Kovac et al., 2021, 2020a). Physical exercise may reduce sleep inertia by targeting key physiological processes of waking that are known to be decreased during sleep inertia (Balkin et al., 2002), specifically by increasing cerebral blood flow and metabolism (Kovac et al., 2021, 2020a; Liu et al., 2023; Renke et al., 2022; Smith and Ainslie, 2017). However, the impact of physical exercise at waking on sleep inertia is still debated. Previous studies testing the effect of physical activity have reported improvements in subjective measures of sleep inertia (Kovac et al., 2021; Kaplan et al., 2018), but either failed to observe significant improvements in objective measures (Kovac et al., 2021) or did not include such measures (Kaplan et al., 2018). Note that Kaplan et al. (2018) tested physical activity in association with other countermeasures such as light exposure and the use of music playlist, while Kovac et al. (2021) used short bouts of exercise of 30 seconds. On the basis this previous evidence we hypothesized that longer activity bouts coupled with breathing exercise could be more effective in decreasing sleep inertia and decrease both subjective and objective measures. On the basis of the evidences about physical activity and respiration, we hypothesized that physical dynamization could be an effective sleep-inertia countermeasure. Physical dynamization is a body movement and breathing protocol initially developed by the French army mental preparation program (ORFA) and proposed to military personnel as a reactivation procedure after sleep, napping or relaxation (Perreaut-Pierre, 2024; Robert and Ravel, 2022).

Given the current literature on reactive countermeasures to sleep inertia, no protocol has yet been clearly identified as effective in dissipating sleep inertia within the first 20 minutes after awakening. Therefore, the aim of this study is to evaluate the effectiveness of two reactive countermeasures — cognitive stimulation and physiological activation (through respiratory and muscular exercises) — in reducing sleep inertia within a controlled setting and a 15-minute time frame. These two countermeasures are compared to a control treatment. Moreover, to assess the generalizability of the countermeasures to different sleeping patterns, the countermeasures were tested both after an unrestrained (9 h of time in bed) night and after a recovery night (3 h of time in bed) occurring after one night of full sleep deprivation.

## 2 Methods

### 2.1 Subjects

42 healthy subjects (18 women and 24 men, < 45 years old), were included in the study. Subjects were free from medical, psychiatric and sleep disorders. Subjects had not travelled between time zones within 7 days prior to the study. Exclusion criteria included physical or mental health troubles based on (I) Hospital Anxiety and Depression scale (HAD < 16), (II) significant medical history, (III) Epworth Sleepiness Scale (ESS > 10), (IV) Pittsburg Sleep Quality Index (PSQI > 8), (V) morningness-eveningness questionnaire < 31 or > 69, or (VI) habitual time in bed per night < 6 hours. Participants were excluded if they self-reported any use of medications with sleep related side effects and illicit drugs. No formal statistical power analysis was performed to determine the sample size; as such, this must be considered a strictly exploratory study that may be underpowered to detect complex statistical interactions or effects of small size. The education level was recorded using the International Standard Classification of Education, ISCED from UNESCO, 1997 version.

The study was conducted in accordance with the principles outlined in the Declaration of Helsinki (1975, revised in 2001). In compliance with French laws, the protocol and procedures received approval from the independent ethics committee EST III (Nancy, France) and were registered with the French National Agency for Medicines and Health Products Safety (ANSM). Written informed consent was obtained from all participants, who received financial gratification for study completion.

### 2.2 Reactivation countermeasures

The study was a randomized, controlled study in parallel arms. The subjects were divided in 3 groups: two active groups with countermeasure and one control group:

#### 2.2.1 Control group

The subjects were awaked by an investigator and the room light was set to 500 lux (white light). The subject had to get up and sit down at the desk. The following sentence was written on the computer’s screen “Regularly touch the computer’s space bar for 5 minutes”. “Press the space bar to start” A 5-minute timer was presented on the screen. This condition presented some of the features of the cognitive stimulation countermeasure (see below) without the cognitive engagement necessary for it.

#### 2.2.2 Cognitive stimulation

The subjects were awaked by an investigator and the room light was set to 500 lux (white light). The subject had to get up and sit down at the desk. The subject had to perform a 5-minute computed Digit Span Memory Test (PsychoPy2, V2024.2.4, www.psychopy.org), a verbal short-term and working memory task, during the 5 minutes following awakening. The Digit Span Test measured their ability to remember a sequence of numbers that appeared on the screen, one at a time. The subject had to memorize a number (from 2 to 6 digits, 1-s stimulus duration) and to write the number on the screen (5-s of retention delay). If they correctly recalled all numbers, then the following sequence was one digit longer. If the participant made an error, then the following sequence was one digit shorter. Research had shown that the digit span task activates brain regions in the frontal cortex (Hampshire et al., 2012; Owen, 2000). In aviation, the digit span test has been used for the evaluation of pilot’s cognitive performance (Arikan et al., 2018; Hardy et al., 2007). In a chronic sleep inertia context, the digit span test has been used to study decreased cognitive function (Draganich and Erdal, 2014; Waterhouse et al., 2007). The digit span test has been shown to mainly activate a fronto-parietal-basal ganglia network (Emch et al., 2019; Vartanian et al., 2022), a network that is also thought to be the underpinning of sustained attention and vigilance (Langner and Eickhoff, 2013; Sarter et al., 2001).

#### 2.2.3 Physiological dynamization

The subjects were awaked by an investigator and the room light was set to 500 lux (white light). The following sentence was written on the computer’s screen: “*Practice the physiological exercise for 5 minutes*”. A staff member started the 5 min timer. The physical movements were recalled on the computer screen “*Sit on your bed*, *make 10 energizing breaths” (0.5 minutes, triangular sequence with inspiration, apnea, forced expiration), “Move your head in all directions (10 movements, 0.5 min), Rotate shoulders, arms and wrists 10 times for each (1 min*). Stand up, *repeat 10 energizing breaths (0.5 min), Move your head in all directions (10 movements, 0.5 min), Turn each shoulder, arm and wrist 10 times (1 min), Perform 10 squats with low extension (1 min) with one triangular breath between each extension”.* The participants were invited to memorize the physiological dynamization sequence 8 days before the study, with a member of the staff. They had to practice the method alone every morning during the week before the experiment.

For all treatments, an experimenter stayed in the room to ensure that participants were compliant with the activities prescribed by their treatment.

### 2.3 Measurements

#### 2.3.1 Sleep Inertia measurements

At the time of writing, there are no direct and specific measurements of sleep inertia. Sleep inertia assessment is performed using indirect measures of sleepiness or fatigue (objective, such as performance to cognitive tasks, or subjective, such as sleepiness questionnaires). Similarly, there is no accepted threshold for declaring the presence of sleep inertia or its severity. The abovementioned indirect measures of sleep inertia are compared to a baseline, and the extent of sleep inertia is measured as deviation from baseline (see for example Evangelista et al., 2022). The absence of Sleep Inertia is inferred when measured variables (e.g., subjective sleepiness) return to baseline level. In the current study we have adopted the same approach. Importantly, because no pre-protocol baseline was collected due to operational and logistical constraints, the 55-minute post-awakening measurement was pre-specified as a within-session reference point to evaluate the relative kinetics of recovery, rather than a true rested baseline.

#### 2.3.2 Subjective sleepiness

The subjects’ self-reported instantaneous level of sleepiness was estimated using the Karolinska Sleepiness Scale (KSS), a widely validated 9- point Likert ranging from “1” (extremely alert) to “9” (extremely sleepy, fighting sleep) (Åkerstedt et al., 2014). KSS is consistently used to follow the kinetic of sleep inertia (Hilditch et al., 2016b). Moreover, high KSS scores have been associated with increased pilot risk taking (Cabon et al., 2012) and is considered as a Risk Fatigue Index (Thomas et al., 2015). Empirically, KSS values equal or greater than 7 are considered as operationally unsafe (Nwaogu et al., 2025; Anderson et al., 2023; EASA, 2019).

#### 2.3.3 Sustained attention

Sustained attention was evaluated using the Psychomotor Vigilance Test (PVT), widely recognized as the gold standard for measuring behavioral alertness in sleep and circadian research (Dinges et al., 1985; Basner et al., 2011). In this task, participants are instructed to respond as rapidly as possible to the appearance of a simple visual stimulus. Reaction Times (RTs) are continuously recorded. The task is time-limited: if no response is registered within 1 second, the stimulus disappears. Similarly, the stimulus is immediately removed upon participant response. Inter-trial intervals are randomly varied between 2 and 10 seconds, and no performance feedback is provided. The PVT protocol administered in this study had a duration of 3 minutes (PVT-B, Basner et al., 2011) that has been already validated in operational contexts (Basner and Rubinstein, 2011; Benderoth et al., 2021; Mollicone et al., 2023).

Two primary outcome measures were derived from the PVT: Reaction Times (RTs) and the number of attentional lapses. Lapses were operationally defined as RTs exceeding 500 ms, while anticipatory responses — RTs shorter than 100 ms — were classified as errors and excluded from the analysis. This study employed a validated and previously developed PVT implementation (Khitrov et al., 2014).

#### 2.3.4 Sleep inertia questionnaire

The Operational Assessment of Sleep Inertia State (OASIS) (Buratto et al., submitted) is a recently developed questionnaire, adapted from the Sleep Inertia Questionnaire (SIQ) (Kanady and Harvey, 2015). The OASIS (**Table 1**) measures sleep inertia as a multi-dimensional concept (cognitive, behavioral and physiological inertia). Buratto and colleagues (submitted) modified the SIQ to assess participants’ experience of the awakening process. The authors rephrased the SIQ instruction to gain instantaneous information about the wake-up process of the experimental morning (rather than trait information from the last week). To simplify further the questionnaire, the authors removed the questions related to emotional inertia, not adapted to an operational context, and transformed the scale response (from 1 to 5) in a dichotomic evaluation (Yes or No). In a validation study, it has been shown that the OASIS may be used as a valid index of sleep inertia, associated with impaired sustained attention and vigilance during the 30 minutes following awakening (Buratto et al., submitted). The OASIS final score is equal to the number of items having received an affirmative answer.

**Table 1:** Operational assessment of sleep inertia state (OASIS).

Answer	Yes	No
1. You had problems getting out of bed		
2. You noticed that it was difficult to keep your balance		
3. You bumped into and/or drop objects		
4. You noticed that your arms and/or legs felt tired and heavy		
5. You noticed that your eyes felt heavy, sore or itchy		
6. You noticed that you move slower than usual		
7. You noticed that your mind felt groggy, fuzzy or hazy		
8. You wish you could sleep more		
9. You notice that you feel sleepy		
10. You feel that you think slower than usual		
11. You feel that you react slower than usual		
12. You have difficulty concentrating		
13. You find that you make more mistakes/errors		
14. You have difficulty getting your thoughts together		

#### 2.3.5 Objective sleep measures

A PolySomnoGraphy (PSG) was performed using a Siesta 802 (Compumedics Limited, Victoria, Australia) with the following ElectroEncephaloGraphic (EGG) derivations: F3/M2, F4/M1, C3/M2, C4/M1, O1/M2, O2/M1; 256 Hz sampling rate with a 0.03–35 Hz bandpass filter; bilateral ElectroOculoGraphic (EOG), ElectroCardioGraphic (ECG), submental and bilateral leg ElectroMyoGraphic (EMG) recordings were also performed. Silver-silver chloride (Ag-AgCl) EEG cup electrodes were applied to the participants’ scalps using EC2 electrode cream (Grass Technologies, Astro-Med, Inc., West Warwick, RI, USA), following the international 10–20 system for electrode placement. Auto-adhesive electrodes (Neuroline 720, Ambu A/S, Ballerup, Denmark) were used for EOG and ECG recordings.

Each PSG records were independently scored by 2 trained and experienced registered sleep technologists following the guidelines of the AASM (Iber, 2007; Iber et al., 2007). to avoid the known biases that can arise from single scorer rating (Collop, 2002; Younes et al., 2016).

Classical macrostructure sleep variables considered in the study were: Time In Bed (TIB; i.e., time from lights off to final awakening, in minutes), Actual Sleep Time (AST, time from the first appearance of N1 to the last sleep epoch, in minutes), Total Sleep Time (TST; i.e., time spent in Non-Rapid Eye Movement [NREM] and Rapid Eye Movement [REM] sleep stages, in minutes), Sleep Onset Latency in minutes (SOL), sleep stages duration (N1, N2, N3, REM) and proportion over TST, duration of Wake After Sleep Onset over TST (WASO) and Sleep Efficiency (SEI%; i.e., percentage of TST over TIB).

#### 2.3.6 Study design and testing conditions

During this protocol, subjects were housed in the laboratory including: 1) a night with a 9 hours’ Time in bed (TIB) (22:00 to 07:00), considered the baseline night, and 2) a sleep deprivation protocol including one night of total sleep deprivation (i.e. 44 hours of continuous awakening), following by a 3 h TIB sleep recovery period (04:00 to 07:00). These two nights were included to be able to compare the effects of the three interventions after normal sleep and after sleep deprivation.

**Figure 1:**
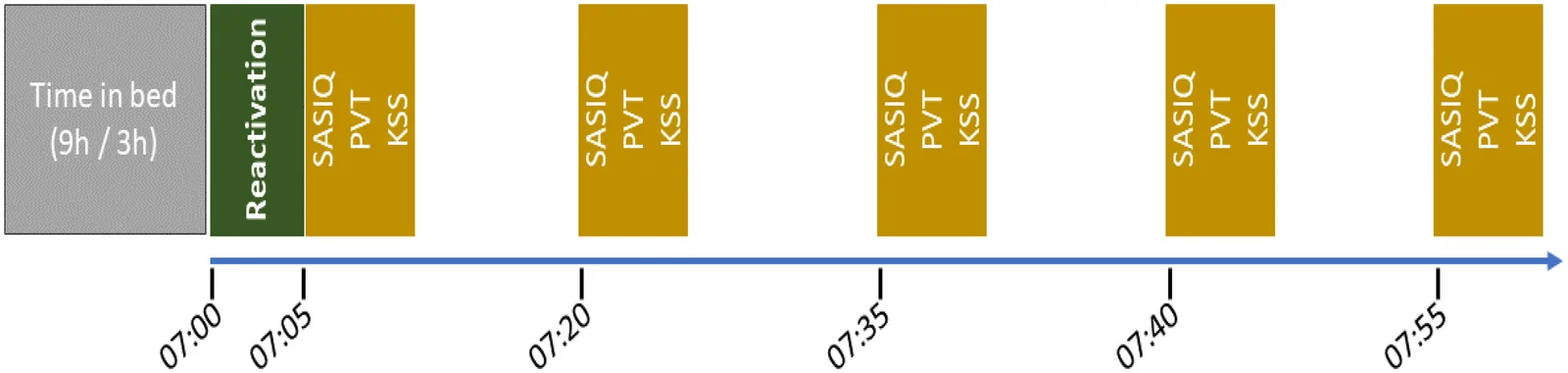
Protocol with the periods of measurements, after 9h time in bed and after 3 hours’ time in bed.

After each awakening the participants practiced their group-specific reactivation countermeasure during 5 min (CTRL, CS or DYN). Measures of subjective sleepiness (KSS), sustained attention (5-minute PVT) and subjective sleep inertia (OASIS) were recorded at 5-min, 20-min, 35-min, 40-min and 55-min after awakening (**Figure 1**).

This study has been conducted in a sleep laboratory. The ambient temperature was controlled and maintained at 22 ± 1 °C during all the experiment. To reproduce controlled lighting conditions, the brightness was maintained à 500 lux in the bedrooms and 200 lux in the living room during the awaking periods. Lights were off during sleep periods. Participants maintained their regular caffeine consumption during the week before the experiment. In the laboratory, they were not allowed to practice exercise, taking tobacco, alcohol, or other psychoactive substances during the study to prevent induced sleep disorders. They were under visual surveillance of research staff members. In addition, we used wrist actigraphy to ensure compliance with the sleep restriction schedule. When participants were not engaged in testing, meals, or sleep periods, they were allowed to read, watch videos, or speak with other participants or staff members and play games, following a pre-established program.

This study was not blinded: the nature of the 3 treatments made so that neither the participants nor the experimenters could be blind to their nature. Note that author performing the analysis was not blinded to the group identity either.

Participants were randomly assigned to one of three groups in a 1:1:1 allocation ratio using a permuted block randomization procedure with a fixed block size of four. Each block was constrained to include at least one participant per group. Randomization was stratified by sex and age to ensure balanced distribution of these prognostic factors across groups. The random allocation sequence was generated using a computer-based random number generator (blockrand package for R software) prior to trial commencement. Participants were enrolled by an investigator and the and group assignment was performed by a data manager responsible for enrolling participants using a sequentially numbered list.

#### 2.3.7 Primary outcome

The primary outcome of the study was the PVT RTs measured 20 minutes after awakening after the recovery night. We considered a treatment effective if participants assigned to that treatment presented shorter RTs 20 minutes after awakening relative to the control group.

#### 2.3.8 Statistical analyses

Statistical analyses were computed using R-studio (V 0.99.902 – 2009–2016 RStudio, 247 Inc.). Values were expressed as mean ± 95% Confidence Interval (95% CI). Groups characteristics were compared using a one-way ANalysis Of VAriance (ANOVA). A mixed linear model (Lmer function, Lme4 library) was used to determine the effect of time (5 levels), TIB (2 levels) and groups (3 levels) on PVT, KSS and OASIS values. In order to take into account the repeated measurements we added a random intercept per subject, and random slopes for TIB and time. Effect sizes were estimated by calculating the eta square (h² > 0.01 indicates a small effect, h² > 0.06 a medium effect and h² > 0.14 a large effect). In the case of significant main effect or interaction, significant differences between conditions were tested using Tukey *post-hoc* tests. Effect size Cohen’s d coefficient was calculated for pairwise comparisons (d> 0.2 indicates a small effect, d > 0.5 a moderate effect and d> 0.8 a large effect).

The kinetics of sleep inertia were assessed by comparing KSS, PVT, and OASIS scores recorded at 5-, 20-, 35-, and 40-minutes post-awakening to those recorded at 55 minutes, which we used as baseline. This reference point was chosen based on the assumption that the majority of the effects of sleep inertia would have been dissipated after 55 minutes of waking, since most studies report that sleep inertia effects are resolved within 30 minutes from awakening (Achermann et al., 1995; Dawson et al., 2021; Ferrara and De Gennaro, 2000; Jewett et al., 1999; Sauvet et al., 2024). While pre-protocol measure would have been a better choice for baseline, we didn’t collect it for logistic reason and used the 55 minutes as an approximate reference point for normal performance levels for our participants. To correct for multiple comparisons, the Benjamini and Hochberg method was applied to control the Type I error rate (Benjamini and Hochberg, 1995). Corrected p-values less than 0.05 were considered statistically significant.

## 3 Results

All 42 enrolled subjects (33.5 ± 1.3 yrs old) completed the protocol. Subjects’ average daily caffeine consumption was 250 ± 32 mg. The average weight was 67.5 ± 0.6 kg, and the average Body Mass Index (BMI) was 23.5 ± 0.6 kg/m². The mean weekly exercise duration was 3.1 ± 0.4 h., the daily TST estimated using actimetry was 7.2 ± 0.2 h and the Epworth sleepiness score was 6.8 ± 0.6. The chronotypes were distributed as follows: 22 subjects were of intermediate chronotype, 12 were of the morning, and 9 was of the evening, without extreme chronotypes. No differences were observed between groups. Seven participants had an upper secondary degree, 12 a post-secondary non-tertiary degree, 7 a short-cycle tertiary degree and 16 a bachelor or equivalent diploma.

During the protocol, the mean daily total sleep time was 8.1 ± 0.3 h during the habitual sleep (9h TIB) condition and 2.8 ± 0.1 h during the sleep restriction condition (3h TIB). The characteristics of the baseline and recovery night are detailed in **Supplementary Table 1a** respectively.

After the baseline night, 47.5% of subjects were awaked in N2 stage, 32.5% in REM stage and 20% were spontaneously awake at 07:00. During the recovery night, N3 sleep stages were the main sleep stages observed during the recovery night (70% for each group). No differences were observed between groups. After sleep restriction, 35.7% of the subjects were awaked in the N3 stage, 28.6% in REM sleep and 26.2% in the N2 stage. Only 2 subjects were spontaneously awake at 07:00.

### 3.1 PVT RTs

For PVT RTs, we observed a significant effect of day (F_1,42_ = 5.2, p=0.001, h² = 0.16) and time of measurement (F_4,42_ = 3.2, p=0.02, h² = 0.14), and a significant three ways interaction (F_12,528_ = 2.5 p=0.04, h² = 0.07) between time, day and group. After the normal sleep night, for the control group, RTs values were higher during the 35 minutes PVT (p=0.03) in comparison to the 55-min values. In the CS group, higher values were observed at 5 (+40 ms, d = 0.7, p=0.03) et 15 min (+ 30 ms, d = 0.6, p=0.03) in comparison to 55 min (**Supplementary Table S2a**). No significant differences were detected across time points for the DYN group. After sleep deprivation and the recovery sleep night, PVT RTs were increased in comparison to baseline night only for the CTRL group (p=0.01 at 05 and 15 min). In the CTRL group, PVT RTs were higher than those recorded after 55 minutes for measures taken at 5 (+ 39 ms, d=0.81, p=0.01) and 15 (+49 ms, d=0.82, p=0.01) minutes after waking up.

In DYN compare to CTRL group, we observed lower RTs after 5 min post awakening (Difference [D] = 49 ms, Cohen’d = 0.81, p=0.02) and 15 min (D = 49 ms, Cohen’d = 0.71, p=0.02). Whereas, in CS compare to CTRL group, RTs were also lower 5 min (D = 20 ms, Cohen’d = 0.39, p=0.03) and 15 min (D = 31 ms, Cohen’d = 0.60, p=0.03) after waking up.

**Figure 2:**
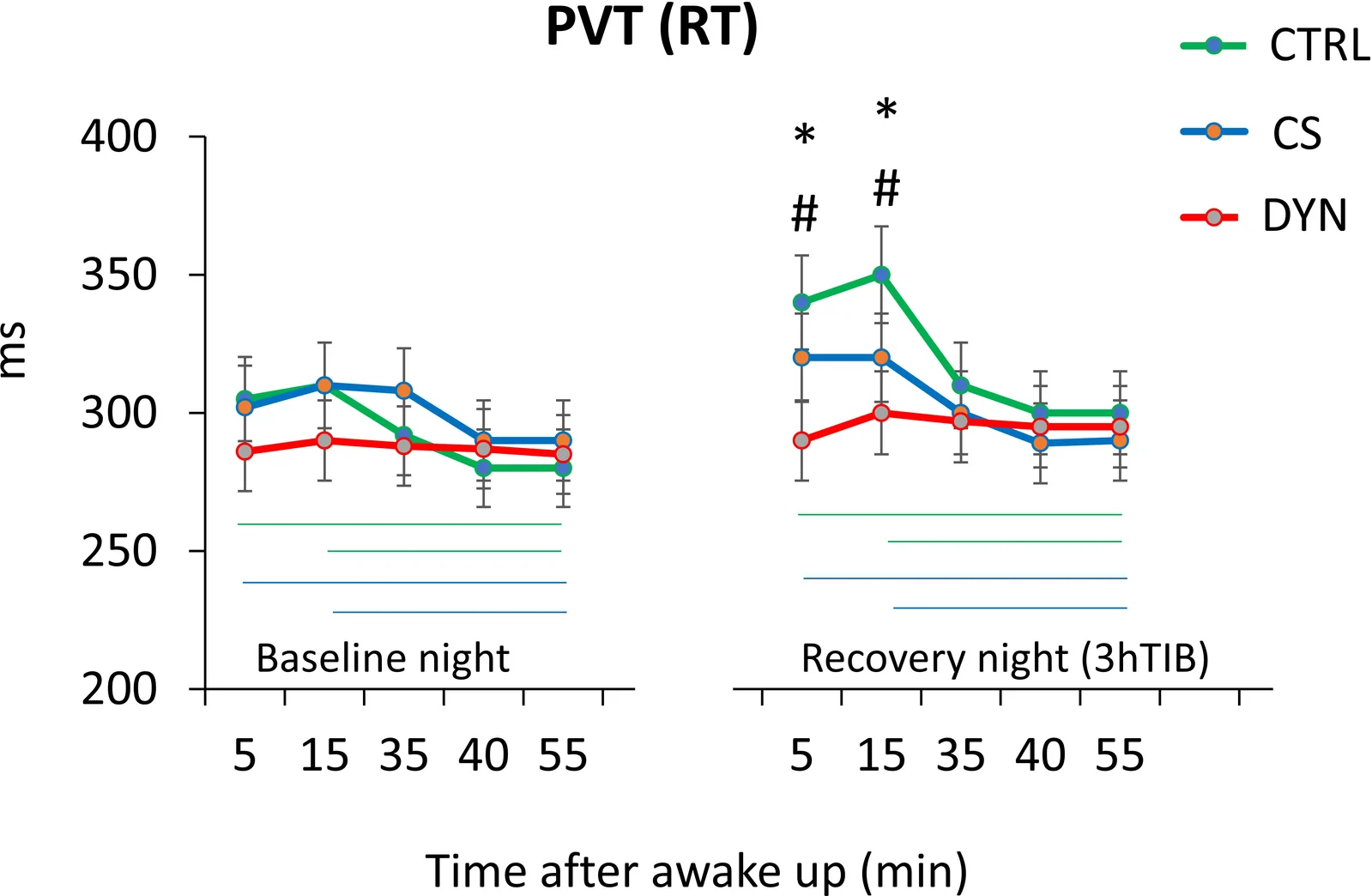
Time course of psychomotor vigilance task (PVT) (reaction time, RTs) values during protocol after baseline (left) and recovery nights (right). *Dynamization (DYN) vs Control (CTRL), # (Cognitive Stimulation) CS vs Control (CTRL), $ Dynamization (DYN) vs (Cognitive Stimulation) CS, p < 0.05. The blue and green lines under the curves represent significant differences relative to the difference with 55 min point. These lines are color coded for group (green, CTRL; blue, CS; red, DYN).

In the CS group, we observed a significant kinetic with increased PVT(RT) values at 5 (+30 ms, d=0.71, p=0.04) and 20 minutes (+0.29 ms, d=0.61, p=0.04) in comparison to 55 minutes. We did not detect a significant difference between 55 minutes and any other measure points for the DYN group. Results are graphically reported in **Figure 2**.

### 3.2 Psychomotor vigilance task - lapses

**Figure 3:**
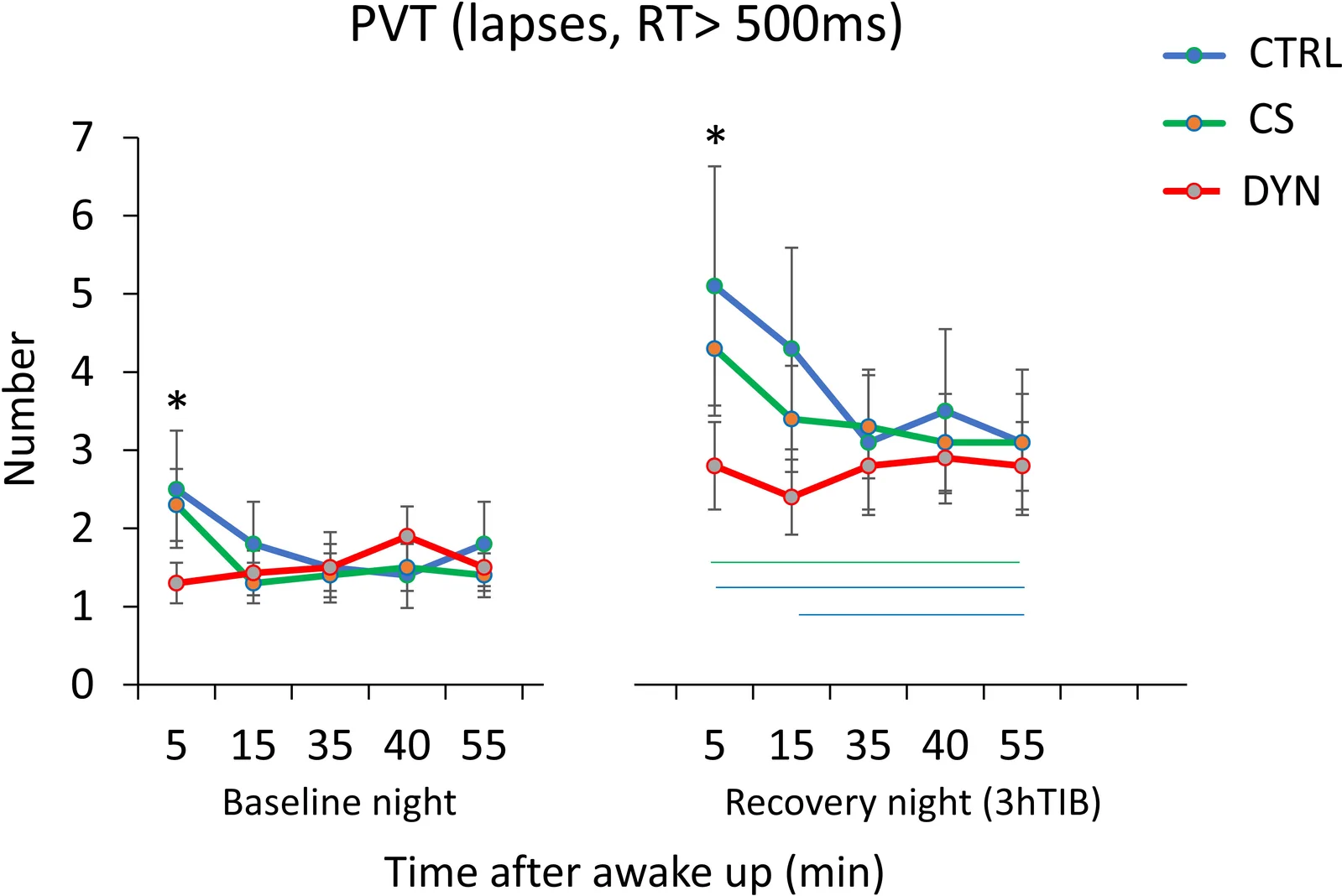
Time course of psychomotor vigilance task (PVT) lapses (RT > 500 ms) during protocol values after baseline (left) and recovery (right) nights. Dynamization (DYN) vs Control (CTRL), # (Cognitive Stimulation) CS vs Control (CTRL), $ Dynamization (DYN) vs (Cognitive Stimulation) CS, p < 0.05 The blue and green lines under the curves represent significant differences relative to the 55 min point. The color code is the same as the main plot. The blue and green lines under the curves are the difference with 55 min point. These lines are color coded for group (green, CTRL; blue, CS; red, DYN).

Considering PVT lapses, we observed a significant effect of day (F_1,42_ = 6.2, p=0.001, h² = 0.16), a significant interaction between time and day (F_4,42_ = 2.2, p=0.04, h² = 0.08), and of the interaction of time, group and day (F_8,528_ = 2.2 p=0.04, h² = 0.08) (**Figure 3**). Participants in the CTRL group showed a higher number of lapses at 5 minutes (+2.0, d=0.61, p = 0.03) and at 20 minutes (+1.3, d=0.56, p = 0.04) relative to the measures taken at 55 minutes after the recovery night (**Supplementary Table S2b**). We did not detect any significant difference among the 5, 15, 35 and 40 timepoints relative to the 55 minutes timepoint for the CS and the DYN group. The number of lapses at the 5 minutes PVT was lower in the DYN group, in comparison to CTRL following the baseline (D = 1.1, d=0.54, p=0.04) and the recovery night (D = 2.3, d=0.65, p=0.04).

### 3.3 Karolinska sleepiness scale

**Figure 4:**
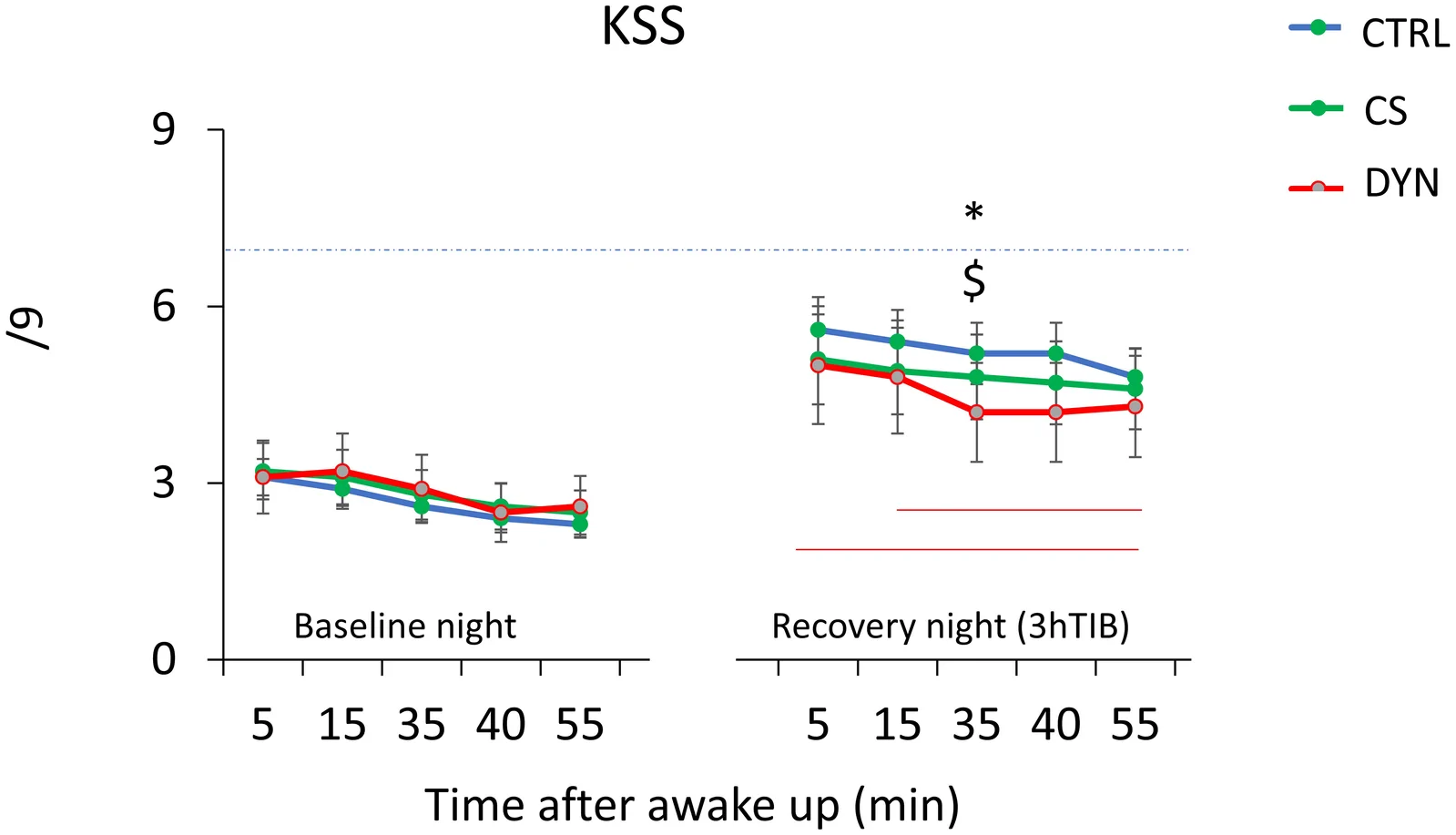
Time course of Karolsinaka sleepiness scale values during protocol after baseline (left) and recovery (right) nights. Dynamization (DYN) vs Control (CTRL), # (Cognitive Stimulation) CS vs Control (CTRL), $ Dynamization (DYN) vs (Cognitive Stimulation) CS, p < 0.05. The red lines under the curves represent significant differences relative to the 55 min point. The dotted line represents a score of 7 - deemed operationally relevant drowsiness by the EASA. These lines are color coded for group (green, CTRL; blue, CS; red, DYN).

For KSS, we observed a significant effect of day (F_1,42_ = 8.2, p=0.001, h² = 0.16), a significant effect of time (F_4,42_ = 2.8, p=0.03, h² = 0.08) and a significant interaction between group, time and day (F_8,528_ = 1.9 p=0.04, h² = 0.07) (**Figure 4**). After the baseline night, all KSS measures were lower (<4) than after the recovery night (p=0.03). After the recovery, we observed lower values of KSS in the DYN group in comparison to CS (-0.6, d = 0.54, p=0.04) and CTRL (-1.0, d=0.62, p=0.02), 35 min after waking (**Supplementary Table S2c**). Moreover, we observed higher KSS values 5 (+0.8, d=0.63, p=0.03) and 15 min (+0.5, d=0.51, p=0.04) after awakening in the DYN group, in comparison to 55 min after awakening. This effect was not observed in the CTRL and CS groups.

### 3.4 Sleep inertia questionnaire OASIS

**Figure 5:**
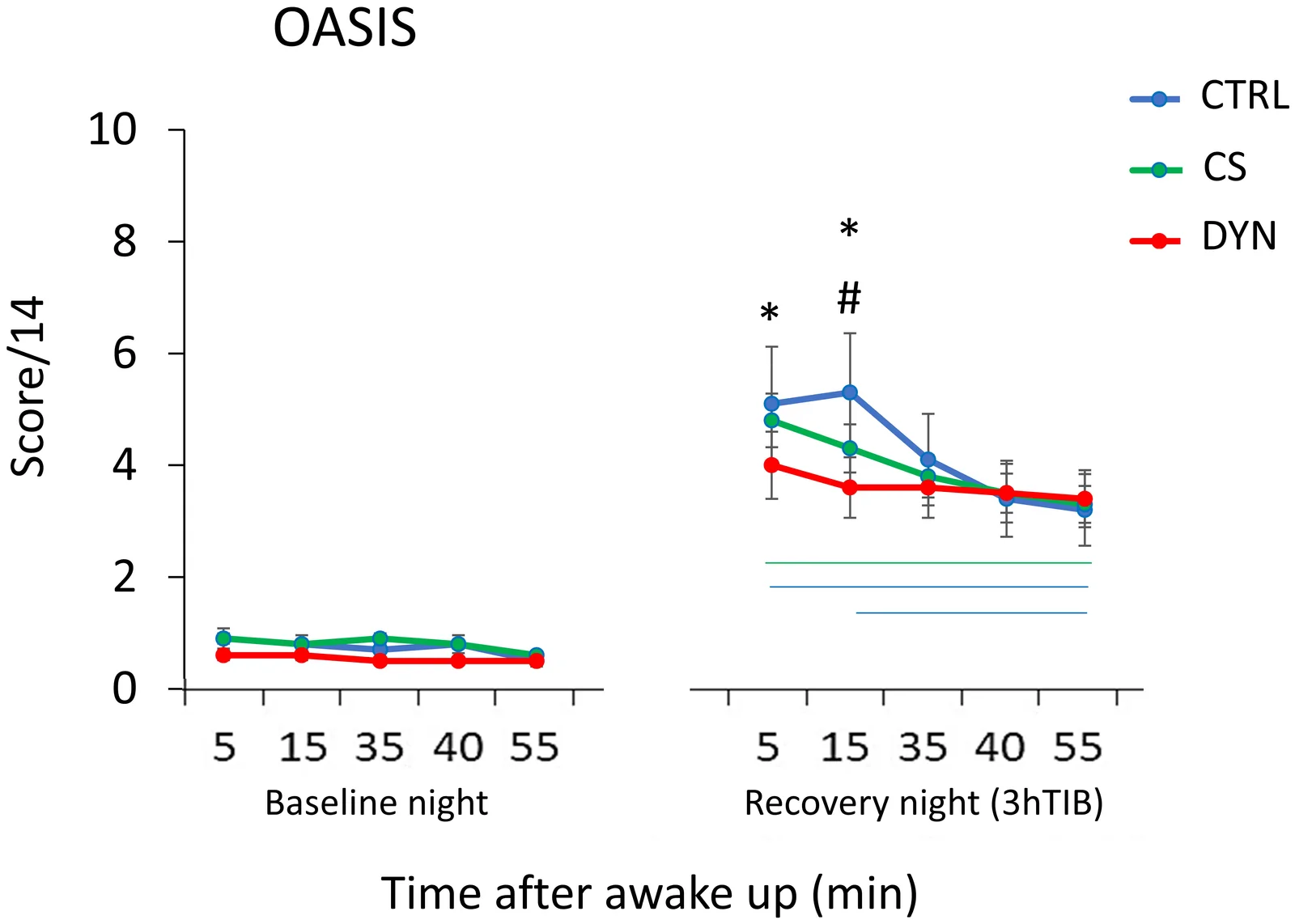
Time course of sleep inertia questionnaire (OASIS) values during protocol after baseline (left) and recovery (right) nights. Legend: *Dynamization (DYN) vs Control (CTRL), # (Cognitive Stimulation) CS vs Control (CTRL), $ Dynamization (DYN) vs (Cognitive Stimulation) CS, p < 0.05 The blue and green lines under the curves represent significant differences relative to the 55 min point. These lines are color coded for group (green, CTRL; blue, CS; red, DYN).

For the sleep inertia questionnaire, we observed a significant effect of day (F_1,42_ = 6.1, p=0.001, h² = 0.14), a significant effect of time (F_4,42_ = 3.1, p=0.03, h² = 0.09) and a significant interaction between group, time and day (F_8,258_ = 2.3 p=0.03, h² = 0.09) (**Figure 5**). After the baseline night, the values were lower than after the recovery night (0.9 ± 0.4 vs 4.2 ± 1.1, p=0.001). After recovery night, we observed a kinetic of ASIQ values for the CTRL and the CS group, with higher values in comparison to 55 min point at 5 (D = 1.1, d=0.61, p=0.03 and D = 0.3, d=0.45, p=0.04) and 20 minutes (D = 1.7, d=0.75, p=0.02 and D = 1.0, d=0.63, p=0.03) after wake up (**Supplementary Table S2d**).

In the DYN group, there was no significant effect of time, and we observed lower values in comparison to CTRL (p=0.04 at 5 min, p=0.03 at 15 min). In the CS group, lower values were observed 15 min after waking (p=0.03).

## 4 Discussion

The protocol was designed to evaluate the effectiveness of cognitive stimulation and physiological dynamization on sleep inertia following either a normal night of sleep (9h TIB) or a partially restricted night of sleep (3h TIB). Previous research has consistently shown that the effects of sleep inertia are exacerbated by prior sleep deprivation (Dinges et al., 1985; Hilditch et al., 2016a, 2017; Miccoli et al., 2008; Rosa et al., 1983; Tassi et al., 2006a). Consistent with these findings, overall poorer performance—reflected by higher PVT reaction times and more PVT lapses—as well as increased subjective sleepiness and sleep inertia—indicated by higher KSS and OASIS scores—were observed after the 3h TIB compared to the 9h TIB. For the first time, we show in our work that physiological dynamization, including respiratory and muscular exercise, can reduce both subjective and objective measures of sleep inertia during the 15 first minutes after awaking. While some authors provided theoretical support for exercise as a potential sleep inertia countermeasure (Kovac et al., 2020a), or shown improvements in subjective measures of sleep inertia (Kovac et al., 2021; Kaplan et al., 2018), we provide empirical evidence of improvement in vigilance, as measured by the PVT, for the first time. As demonstrated by Kovac et al. (2020a), even short bouts of movement upon waking (e.g., 30 seconds) are sufficient to activate sympathetic nervous system activity in healthy individuals during night-shift conditions. Compared to sedentary controls, exercise was shown to reduce subjective sleepiness and induce a temporary increase in sympathetic activity, as reflected by elevated plasma noradrenaline levels. Exercise has been investigated as a potential countermeasure due to its ability to stimulate the key physiological processes associated with the transition from sleep to wakefulness., including the reactivation and deactivation of various brain regions and changes in cerebral blood flow (CBF) (Balkin et al., 2002), the cortisol awakening response (Clow et al., 2010), and thermoregulatory processes, notably the increase in core body temperature and cooling of extremities (Krauchi et al., 2006). Aerobic exercise in particular, is well known to affect all the above-mentioned processes (Anderson and Wideman, 2017; Kovac et al., 2020b).

In our work we showed a stronger effect of dynamization on sleep inertia compared to cognitive stimulation. We did not detect a significant effect of time on sleep inertia for the dynamization group for the PVT indices and for subjective sleep inertia (OASIS). While this could be related to a total lack of sleep inertia for this group, it cannot be excluded that the lack of a significant effect is related to a lack of statistical power caused by the limited sample size, rather than to a lack of sleep inertia. Notice that when considering our primary outcome (PVT RTs 20 minutes after awakening) only dynamization can be considered effective. Since no previous studies compared physical dynamization with cognitive stimulation it is not obvious to pinpoint the possible causes of the differences observed for the two treatments. One possible explanation is that cognitive stimulation only has a domain-specific effect, in that it reactivates the specific cerebral network associated with the forward digit span task, that is a fronto-parietal- basal ganglia network (Emch et al., 2019; Vartanian et al., 2022). On the other hand, physical activity has been shown to increase cerebral blood flow and metabolism globally (Balkin et al., 2002; Anderson and Wideman, 2017), and to increase cortisol level (Clow et al., 2010), hence exerting a holistic effect on the brain. Future research may elucidate the observed differences by employing electrophysiological or imaging methods to observe possible differential effects of the treatment on brain activity, or by using an integrated cognitive stimulation approach, involving multi-domain tasks, to reactivate multiple brain networks., Finally, cortisol measurements may be integrated in future study to assess the possible differential involvement of stress hormone dynamics in the effects of cognitive stimulation and dynamization (Kovac et al., 2020a).

Indeed, the effect of cognitive stimulation was not as clear cut as that of dynamization. Indeed, participants in the cognitive stimulation group had results compatible with a shorter sleep inertia duration for the PVT lapses and OASIS, relative to the control group. However, they showed the same pattern of results than the control group for PVT RTs. Few studies have previously evaluated the interest of cognitive stimulation alone as a countermeasure to sleep inertia. There was no evidence that a high cognitive stimulation could decrease the amplitude of the kinetics of sleep inertia, in comparison to a low cognitive demand situation. In 23 young healthy men, after 7.5 h of sleep, Kovac and colleagues (2020) observed that anticipating a stressful task before sleep reduces the psychomotor vigilance test and Karolinska sleepiness scale amplitude of sleep inertia (Kovac et al., 2020b). The impact of cognitive stimulation on sleep inertia needs further laboratory and field studies. It has been observed that cognitive stimulation using a loud and sharp sound and flashing lights may reduce the severity of sleep inertia (Wörle et al., 2020). However, in this study, the same sleep inertia reduction was observed with a comfortable and pleasant wake up with soft music and a warm yellow light and no differences were observed between the two strategies.

Overall, our findings are in line with previous studies showing that a short (i.e., 3 hours) recovery period following a sleep deprivation is associated with an increased risk of sleep inertia (Hilditch et al., 2016b, 2017). Most of the participants in the current study have been awakened from N3 sleep following the restricted night, thus increasing the probability of observing sleep inertia (Hilditch and McHill, 2019; Trotti, 2017).

Significant time-dependent differences were predominantly observed at 5- and 15-minutes post-awakening compared to the 55-minute mark, both in terms of objective performance measures and subjective assessments such as the Karolinska Sleepiness Scale (KSS) and the Sleep Inertia Questionnaire (OASIS). This suggests that the dissipation of sleep inertia reaches a plateau at approximately 35 minutes after awakening. Nevertheless, subjective sleepiness values remained higher after sleep restriction than after the baseline night, even at the 55 minutes mark. Most prior studies on sleep inertia were not specifically designed to assess its duration, and therefore included a limited number of repeated measurements, limiting conclusions regarding the temporal dynamics and the contributing factors to sleep inertia. Studies comparing performance after sleep inertia to pre-sleep values have typically shown a return to these levels within 30 min of awakening (Brooks and Lack, 2006; Salamé et al., 1995; Tietzel and Lack, 2001; Sauvet et al., 2024) and sometimes as soon as 20 or 15 min after awakening (Kovac et al., 2021; Scheer et al., 2008). Studies that have systematically measured alertness and performance across the period after waking, however, report an asymptotic dissipation of sleep inertia (Hilditch and McHill, 2019). Achermann et al. (1995) suggested that although initial recovery is rapid, complete dissipation of impairment may take up to an hour. Jewett et al. (1999) similarly reported that performance deficits are greatest immediately upon awakening, with recovery occurring between 15 and 60 minutes. It is important to note that these studies were conducted in highly controlled laboratory environments, and the kinetics of sleep inertia may differ in real-world, high-demand operational settings.

In the current study, we observed differences between objective and subjective sleep inertia trajectories. These results are consistent with previous findings that subjective ratings of sleepiness may be inconsistent predictors of objective performance under conditions of partial and chronic sleep loss. For example, Achermann et al. (1995) emphasized that subjective measures may fail to track the true time course of performance recovery. Similarly, Hilditch and McHill (2019) found that participants subjectively rated their performance as improved after waking from a 30-minute nighttime nap, despite objective attentional impairments. In the current study, the kinetic pattern of the OASIS closely paralleled objective performance measures, in contrast to the KSS. These findings suggest that the OASIS may represent a more accurate self-assessment tool for monitoring sleep inertia when objective performance measures are not available.

### 4.1 Limitations

Several key limitations must be acknowledged. First, the small sample size of 14 healthy participants per group underscores the purely exploratory nature of this trial. Consequently, the study is likely underpowered to detect complex higher-order statistical interactions or smaller localized group differences, limiting the definitive nature of our observations. Future studies with larger, more heterogeneous samples are needed to strengthen the validity and applicability of results. Furthermore, this investigation was conducted in a controlled laboratory environment following a brief sleep period after total sleep deprivation. In real-world operational contexts such as aviation and long-haul transport, chronic sleep restriction and associated sleep disorders frequently occur and may intensify sleep inertia effects (Balkin and Badia, 1988; McHill et al., 2014). These findings, therefore, represent potential implications rather than direct operational proof. Future field studies using realistic operational tasks and true performance endpoints are required to determine whether physiological dynamization provides authentic functional benefits in safety-critical settings like flight decks. Related to this, it should be noticed that while we used a validated and widely used sustained attention task as an objective measure of sleep inertia, the PVT is far removed from an operational task. Future study should include simulated operational tasks to characterize the functional impact of sleep inertia and the effectiveness of countermeasures for real world operations.

Neither the participants nor the experimenters were blinded to the active interventions, and the primary analysis was similarly unblinded. This lack of blinding introduces a clear risk of expectation bias, which may have inadvertently influenced the subjective metrics (such as the KSS and OASIS) and potentially skewed the behavioral responses. While the fact that our objective, computerized PVT trends generally mirror these subjective outcomes provides partial mitigation, an expectation effect cannot be entirely ruled out. Devising a viable placebo treatment for sleep inertia for future studies may remove this bias. Another limitation related to out protocol is a lack of quantification of the intensity of the dynamization exercises (e.g., heart rate, or a subjective scale such as the rating of perceived exertion). Future study should include such measures to improve replicability and test for possible effects of the intensity of the effort.

We used the measures taken 55 minutes after awakening as baseline, based on several studies reporting that the majority of sleep inertia effect disappear within 30 minutes from awakening (Brooks and Lack, 2006; Salamé et al., 1995; Tietzel and Lack, 2001; Sauvet et al., 2024). However, because a true pre-protocol rested baseline was not collected, our reliance on this 55-minute within-session reference point prevents us from determining whether absolute baseline performance was ever truly recovered, meaning the outcomes reflect relative within-session dissipation kinetics rather than an absolute baseline return. Future studies should include pre-protocol baseline measures in order to avoid this possible confounding.

Although we have shown that sleep inertia effects can be reduced, and its tapering off accelerated, by physiological dynamization, it is not possible to comment on its reduction in severity from an operational viewpoint. This is because at the time of writing there is no accepted threshold for declaring the presence of sleep inertia or its severity. A suggestion can be made that, overall, the sleep inertia induced by our protocol was only moderate. Indeed, none of our participants reported KSS values (a measure of sleepiness and thus an indirect measure of sleep inertia) equal or higher than 7. This is the threshold used by the EASA to indicate operationally relevant sleepiness (EASA, 2019). However, this is a common occurrence in sleep inertia studies involving healthy participants (see for example Santhi et al., 2013). As for the extent of RTs and lapses increase, they are comparable with what has been reported in previous papers focusing on healthy participants (Santhi et al., 2013) and patients (Evangelista et al., 2022).

The current study examined sleep inertia through a mix of objective and subjective measures. However, the scope of physiological and cognitive indicators was limited. Future research should expand these indicators to achieve a more comprehensive evaluation of countermeasure efficacy. Additionally, while this study tested only cognitive (i.e. working memory) and physiological stimulation as countermeasures, exploring a wider range of interventions, including combined cognitive and physiological methods and diverse cognitive domains such as verbal recall or problem-solving—may enhance understanding.

### 4.2 Conclusion

Physiological dynamization and, to a lesser extent, cognitive stimulation appears to be promising interventions for mitigating the effects of sleep inertia after sleep deprivation. By engaging in respiratory and physical exercise tasks shortly after awakening, individuals can experience faster cognitive recovery and enhanced alertness, with a larger effect than cognitive stimulation. By incorporating reactive countermeasures into wake-up routines, results show that it is possible to accelerate the recovery of cognitive performance. While these findings suggest potential value for inclusion in the wake-up routines of shift workers, emergency responders, or aircraft pilots, additional ecological field testing with authentic operational tasks is vital to establish real-world efficacy and safety benefits.

## Data Availability

The raw data supporting the conclusions of this article will be made available by the authors, without undue reservation.
