# Supplementary material for: Evaluate the effectiveness of cognitive stimulation and physiological dynamization on sleep inertia
Source: Front Physiol. 2026 Aug 24;17:1855408. doi: 10.3389/fphys.2026.1855408 (PMC13548842; doi:10.3389/fphys.2026.1855408)
Supplement: Supplementary file 1 [file Table1.docx]

Evaluate the effectiveness of cognitive stimulation and physiological dynamization on sleep inertia

Beauchamps Vincent, Sauvet Fabien, Boyer Stanislas, Nemmi Federico, Buratto Florence

**SUPPLEMENTARY DATA**

**Table S1 a. Baseline night sleep characteristics**

|  | CTRL | CS | SYN | Anova  F_2, 14_ (p) |
| --- | --- | --- | --- | --- |
| TST, min | 478.69 ± 12.81 | 483.38 ± 11.23 | 481.69 ± 13.47 | 0.34 (0.12) |
| SOL, min | 22.58 ± 4.02 | 26.36 ± 4.21 | 25.81 ± 4.02 | 0.23 (0.87) |
| N1, min | 31.38 ± 8.12 | 52.28 ± 12.36 | 38.64 ± 9.22 | 0.31 (0.58) |
| N2, min | 212.85 ± 14.44 | 221.75 ± 17.35 | 215.33 ± 18.54 | 0.85 (0.34) |
| N3, min | 114.00 ± 24.00 | 108.10 ± 31.21 | 112.20 ± 28.32 | 0.20 (0.67) |
| REM, min | 120.10 ± 5.18 | 102.20 ± 9.32 | 116.43 ± 9.21 | 0.82 (0.44) |
| WASO, min | 32.31 ± 4.54 | 45.24 ± 2.65 | 39.43 ± 3.43 | 0.76 (0.31) |
| Awakening stages, n (%) | |  |  |  |
| N2 | 7 (63.4%) | 7 (58.3%) | 5 (41.6%) |  |
| REM | 4 (28.5%) | 4 (33.3%) | 5 (41.6%) |  |
| Wake | 3 (21.4%) | 2 (16.6%) | 3 (25.0%) |  |

**Table S1 b. Recovery night sleep characteristics**

|  | CTRL | CS | SYN | Anova  F_2, 14_ (p) |
| --- | --- | --- | --- | --- |
| TST, min | 171.1 ± 10.1 | 169.5 ± 15.1 | 170.4 ± 15.5 | 1.4 (0.12) |
| SOL, min | 4.1 ± 6.1 | 4.2 ± 7.1 | 5.0 ± 6.9 | 0.19 (0.66) |
| N1, min | 7.2 ± 10.1 | 5.2 ± 10.1 | 6.8 ± 7.0 | 0.92 (0.34) |
| N2, min | 30.2 ± 15.3 | 28.1 ± 11.1 | 31.2 ± 12.9 | 1.66 (0.21) |
| N3, min | 118.4 ± 24.2 | 117.6 ± 20.1 | 115.3 ± 22.2 | 2.03 (0.16) |
| REM, min | 16.2 ± 10.1 | 17.4 ± 8.1 | 17.1 ± 9.2 | 0.31 (0.58) |
| WASO, min | 5.1 ± 3.1 | 5.7 ± 3.1 | 5.1 ± 5.1 | 0.86 (0.34) |
| Awakening stage, n (%) | |  |  |  |
| N2 | 3 (21.4%) | 4 (28.6%) | 4 (28.6%) |  |
| N3 | 6 (42.8%) | 5 (35.7%) | 4 (28.6%) |  |
| REM | 4 (28.6%) | 3 (21.4%) | 5 (35.7%) |  |
| Wake | 1 (7.1%) | 1 (7.1%) | 0 |  |

CTRL is the control group, CS is cognitive stimulation, PD is physical dynamization

**Table S2a. PVT Lapses**

|  |  |  |  | **CTRL - CS** | | **CTRL-DYN** | |
| --- | --- | --- | --- | --- | --- | --- | --- |
| **Time** | **CTRL** | **CS** | **DYN** | **D** | **d** | **D** | **d** |
| 5 | 5.1 ± 1.5 | 4.3 ± 1.3 | 2.8 ± 0.8 | 0.8 | 0.3 | 2.3 | 0.7 |
| 15 | 4.3 ±1.3 | 3.4 ± 1.0 | 2.4 ± 0.7 | 0.9 | 0.4 | 1.9 | 0.7 |
| 35 | 3.3 ± 0.9 | 3.3 ± 0.1 | 2.8 ± 0.8 | 0.1 | 0.0 | 0.5 | 0.3 |
| 40 | 3.5 ± 1.1 | 2.9 ± 0.9 | 2.9 ± 0.8 | 0.6 | 0.3 | 0.6 | 0.3 |
| 45 | 3.1 ± 0.9 | 3.1 ± 0.9 | 2.8 ± 0.8 | 0.1 | 0.0 | 0.3 | 0.2 |

**Table S2b. PVT RT**

|  |  |  |  | **CTRL - CS** | | **CTRL-DYN** | |
| --- | --- | --- | --- | --- | --- | --- | --- |
| **Time** | **CTRL** | **CS** | **DYN** | **D** | **d** | **D** | **d** |
| 5 | 340 ± 17 | 320 ± 16 | 291 ± 15 | 20 | 0.4 | 49 | 0.8 |
| 15 | 350 ± 18 | 319 ± 16 | 302 ± 15 | 31 | 0.6 | 48 | 0.8 |
| 35 | 310 ± 15 | 302 ± 15 | 297 ± 15 | 8 | 0.1 | 13 | 0.2 |
| 40 | 300 ± 15 | 295 ± 15 | 294 ± 15 | 5 | 0.1 | 6 | 0.1 |
| 45 | 301 ± 15 | 290 ± 14 | 293 ± 14 | 11 | 0.2 | 8 | 0.1 |

**Table S2c. KSS**

|  |  |  |  | **CTRL - CS** | | **CTRL-DYN** | |
| --- | --- | --- | --- | --- | --- | --- | --- |
| **Time** | **CTRL** | **CS** | **DYN** | **D** | **d** | **D** | **d** |
| 5 | 5.6 ± 0.84 | 5.0 ± 0.8 | 5.1 ± 0.8 | 0.6 | 0.4 | 0.6 | 0.4 |
| 15 | 5.4 ± 0.81 | 4.8 ± 0.7 | 4.8 ± 0.7 | 0.6 | 0.4 | 0.6 | 0.4 |
| 35 | 5.2 ± 0.78 | 4.8 ± 0.7 | 4.2 ± 0.6 | 0.4 | 0.3 | 1.0 | 0.6 |
| 40 | 5.2 ± 0.78 | 4.7 ± 0.7 | 4.2 ± 0.6 | 0.5 | 0.3 | 1.0 | 0.6 |
| 45 | 4.8 ± 0.72 | 4.3 ± 0.6 | 4.3 ± 0.6 | 0.5 | 0.4 | 0.5 | 0.4 |

**Table S3d. OASIS**

|  |  |  |  | **CTRL - CS** | | **CTRL-DYN** | |
| --- | --- | --- | --- | --- | --- | --- | --- |
| **Time** | **CTRL** | **CS** | **DYN** | **D** | **d** | **D** | **d** |
| 5 | 5.1 ± 1.5 | 4.8 ± 1.4 | 4.1 ± 1.2 | 0.3 | 0.1 | 1.1 | 0.4 |
| 15 | 5.3 ± 1.5 | 4.3 ± 1.3 | 3.6 ± 1.1 | 1.0 | 0.3 | 1.7 | 0.5 |
| 35 | 3.8 ± 1.1 | 3.8 ± 1.1 | 3.6 ± 1.1 | 0.1 | 0.0 | 0.2 | 0.1 |
| 40 | 3.4 ± 1.1 | 3.5 ± 1.1 | 3.5 ± 1.1 | -0.1 | -0.1 | -0.1 | -0.1 |
| 45 | 3.4 ± 1.0 | 3.4 ± 1.0 | 3.4 ± 1.0 | 0.1 | 0.0 | 0 | 0.0 |

***Tables S2 (a to d) changes in PVT RT, Lapses, KSS and OASIS score in the 3 conditions after TSD. “D” is the difference between means and “d” the Cohen coefficient. Values are mean ± 95%*** *CI*
